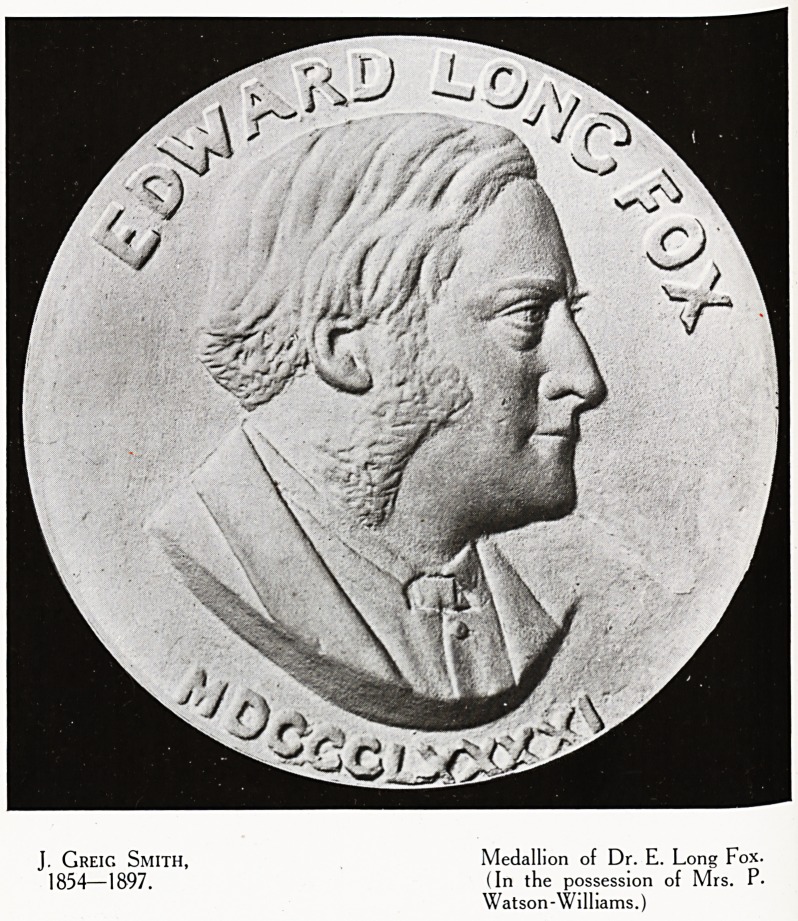# The Debt of Medicine to the Fine Arts

**Published:** 1923-01

**Authors:** J. A. Nixon

**Affiliations:** Physician to the Bristol Royal Infirmary, Consulting Physician to Southmead Infirmary


Leonardo da Vinci,

1452–1519.

*Anatomy of the Muscles.*

### The Bristol Medico-Chirurgical Journal

"Scire est nescire, nisi id me
Scire alius sciret.

JANUARY, 1923.

### THE DEBT OF MEDICINE TO THE FINE ARTS.

The Presidential Address, delivered on October 11th, 1922, at the opening of the Fiftieth Session of the Bristol Medico-Chirurgical Society.

BY

J. A. Nixon, C.M.G., M.D. Cantab., F.R.C.P.,

*Physician to the Bristol Royal Infirmary, Consulting Physician to Southmead Infirmary.*

---

The subject of this address was suggested by a remark in a paper read at the International Congress of Medicine in 1913. Dr. Corson was discussing the artistic powers of Sir Charles Bell, "who," he said, "thought anatomy of great use to the artist." Then he added, "But a great book is yet to be written on the value of art to anatomy and medicine." Although the book has not been written, the evidences of this value of art to medicine are scattered freely through the whole of its history, and there can be no gainsaying that the fine arts have played an important part in the general advance of medical knowledge and in the progress of what is called science. I had often wondered whether there was not some almost miraculous inspiration as yet unaccounted for that caused the evolution of scientific medicine from the welter of superstition and magic in which the healing art originated.

From all we learn by the study of medicine as practised among primitive tribes, or by the study of its earliest traces in prehistoric times even into the dawn of history, it is clear that nothing approaching a scientific spirit existed in medicine before the era of the Greek philosophers. Medicine before the golden age of Pericles was little more than a medley of wonder-working and miracle-mongering practised on a superstitious public by a caste of priestly quacks.

Philosophy began the work of freeing medicine from the delusions of superstition, but it failed in a large measure because it substituted errors of hypothesis in their place. All the real progress in medicine has been due to men who, being by nature richly endowed, like artists, with keen gifts of perception, have retained these gifts undulled, together with the Hippocratic secret of vivid mental imagery.

Hippocrates infused a wholly new spirit into medicine when he discarded both superstition and hypothesis, introducing in their stead the results of actual observation. Khalif Ali, the son-in-law of Mahomet, said that "men are more like the times in which they live than they are like their fathers." It is to the times in which he lived that we must look for an explanation of Hippocrates' supreme gift to medicine. Had he lived in an environment of fanaticism and ignorance, the Father of Medicine might have dealt, like his forerunners and contemporaries, in therapeutic magic and spells.

But the Greeks of the fifth century B.C. were distinguished by intellectual acuteness and artistic gifts, a fertile combination for the growth of science. Hellas possessed men of a new sort. Hitherto superstition and fear, going hand in hand had sufficed to prevent natural curiosity from exploring the paths of scientific enquiry into natural phenomena. Curiosity unaided by acuity of perception remains timid and prone to accept supernatural explanations of the phenomena it witnesses.

"Progress was at last made in the art of medicine because the physicians of Greece shared with her poets and sculptors the same splendid faculties of keen sight and faithful reproduction of the thing seen." Flinders Petrie has pointed out that in successive civilisations there has been a fairly regular sequence in the development of the various branches of human activity. Art appears first, notably sculpture and architecture, literature follows later, and last of all science.

As we read the Hippocratic writings we realise that the acuity of observation that Praxiteles and Pheidias brought to their masterpieces of sculpture finds expression in the Hippocratic description of clinical cases. The theories of Hippocrates were admittedly as speculative and erroneous as any, but when he confines himself to simple observation of patients and their behaviour the artistic perception breaks out. He had a descriptive faculty which has never been approached for excellence, he saw with the eye of an artist. Who but an artist could have described approaching death as he saw it: "A face with sharp nose, hollow eyes, sunken temples; the ears cold, contracted, and their lobes turned out; the skin of the forehead rough, distended, parched; the colour of the whole face being green, black, livid or lead-coloured." Hippocrates had the good fortune to be living in the age of Pericles, one of those rare epochs when "artists, philosophers and men of action cease to live in isolation, but breathe a common air and catch light and heat from each other's thoughts."

As the light of Greece grew dim the intellectual centre changed to Alexandria, where science and medicine, if they progressed at all, did so in the Hellenistic colony in Egypt. But the progress made was small, just as we find that art in their hands soon became stationary and decadent.

In Rome, too, all that survived of science and medicine was a mere thread of Greek tradition. Roman art was in the main degenerate Greek art so far as concerned the plastic and æsthetic arts. Rome was interested chiefly in the arts of government and war, the arts of peace were subjugated under her iron sway.

To Egypt and Rome succeeded the Arabian, Mohammedan or Saracenic dominion. We know that the Arabians preserved for coming ages the literature of Greek medicine. But the Greek spirit was dead, they contributed nothing to the advancement of medical knowledge. Rhazes alone of them, perhaps, observed originally and gave us measles and small pox. Their own writer, Ibn Khaldun, summed them up: "Of all the people in the world they are the least capable of ruling a kingdom, of all the people in the world they have the least aptitude for the arts."

Medicine, in fact, stood still until the fourteenth century, when the remarkable revival of learning in Europe took place. This revival corresponded with (I will not say was caused by) a re-introduction of Greek literature and learning.

Voltaire attributed the rising genius of the Italians to their new-found wealth and liberty, pointing out that before the remains of Greek learning removed from Constantinople into Italy Brunelleschi began to revive the ancient taste in architecture, Giotto brought the freshness of the moderns combined with the vigour of the ancients into painting, Boccaccio established the Italian language, and Guido of Arezzo invented the new method of musical notes. He would not admit that it was to the refugees of Constantinople we are indebted for the "resurrection of letters." "Those men," he says scornfully, "were capable of teaching the Italians nothing more than the Greek tongue."

It is hard to imagine that the last Greeks of Byzantium were really capable of arousing a sudden flood of genius in Western Europe. Nor is it true that the essentials of the Hippocratic treatises had ever been lost. They were extant and uncorrupted throughout the whole of the Saracen dominions; that is to say, the texts were extant, but the spirit was lost.

I think the Renaissance did not revive medicine by restoring Greek writings. If the Greek classics restored anything it was the feeling for the æsthetic arts, but this feeling had already come into being.

Had the master minds of the Renaissance confined their studies to Greek history alone they would have found it a lamentable record of petty strife and treachery, redeemed here and there by some noble and heroic action, but they would have learnt little of the marvellous achievements of the Greeks in the realms of thought and art. "What," asks Professor Andrewes, "does the Peloponnesian War matter in comparison with the invention of mathematics, the development of the drama, or the idealism of Greek sculpture?" The Middle Ages were dark not because the history of Greece had been forgotten but because the art and culture of classical times had been lost. When Europe began to shake off the fetters of ignorance art was the first fruit of the revival. Sculpture and architecture were almost at their zenith in the thirteenth century. Painting lagged nearly two centuries behind. Close on the heels of painting came anatomy, the first of the sciences to free itself from the bondage of antiquity.

The study of anatomy by actual dissection and first-hand observation began indeed with Mondini, who died in

1318. His work was devoid of illustrations or references to diagrams.

Henri de Mondeville is said by Guy de Chauliac (one of his pupils) to have illustrated his demonstrations of anatomy by pictures or diagrams thirteen in number.

When we reach the fifteenth century, however, we are suddenly confronted by the mighty genius of Leonardo da Vinci (born 1452). Leonardo not only studied the anatomy of the human figure for the improvement of his art as painter and sculptor, he brought to the study of anatomy his unrivalled perception as an artist. He not only collaborated with the anatomist Della Torre by illustrating the celebrated anatomical treatise of the latter, but wrote on anatomy from his own observations. In Windsor Castle is a MS. of his anatomical drawings with the explanatory text written in the singular "mirror-writing" that he affected. This is part of a purchase made for King Charles I., possibly acquired for him by the sound judgment of William Harvey. Amongst these are a series of drawings of the bones and muscles, to which he added analytical diagrams for the demonstration of muscular action. (Plate I.)

Leonardo's Windsor MS. was thus described by Robert Knox in 1852 :—

"It is a small folio, prepared as a sketch book, its leaves filled with figures drawn by Leonardo, chiefly from dissections either made by himself or conjointly with Della Torre . . . in no instance could I perceive that Da Vinci ever mistook the dead for the living. As if to secure himself against the possibility of such an occurrence, he has drawn generally, and with a grace and spirit not to be surpassed, the living limb, with all its glorious exterior side by side with the dead and dissected corse. He draws the dead as dead—the living as living. . . . I stumbled on a drawing of the semilunar valves of the aorta in a variety of positions, so

Leonardo da Vinci.

1452–1519.

From the *Windsor MS.*

Plate II.

as to show their descriptive anatomy and their physiological action. The corpuscules of Arantius have not been forgotten. Now all this occurred long before the age of Fabricius and Harvey: and even before that of Vesalius." (Plate II.)

Unfortunately, the work of Leonardo lay forgotten or lost, so that his influence upon medical science was negligible. None of the books on anatomy, surgery or medicine which belong to the period just after Leonardo's show either anatomical accuracy or artistic merit, although the period was one in which artistic talent abounded.

At the date of Leonardo's death, in 1519, Raphael was approaching the end of his labours (he died in 1520); Michelangelo, however, was living, and continued his great work till his death in 1564; Benvenuto Cellini was born in 1500 and died in 1571. This was an age of marvellous intellects—Erasmus, Thomas More, Copernicus, and Luther. Columbus, too, had just discovered America (1492).

Yet medical science seemed to derive little profit, unless the foundation of a College of Physicians in London by Linacre or the establishment of lectures on human anatomy in Cambridge by John Caius should be ranked as events of first importance. The best-known name of this period is that of the half-crazy charlatan Paracelsus, on whom the mantle of Leonardo most emphatically did not fall.

Nevertheless, the barren period was a short one; even at the time of Leonardo's death the boy Vesalius was five years old. His was to prove the master-mind for which was reserved the study of human anatomy based upon direct observation. To turn to the pages of the *De Corporis Humani Fabrica* of Vesalius is to step from the confusing glimmer of conjecture and tradition into clear light and order. From the first this great teacher availed himself of the assistance of the painter and engraver. He not only selected artists of the highest capacity, but himself superintended and directed all their interpretations. To realise how far he out-distanced his predecessors we may compare a plate from the anatomy of Phries (1518) with one from Vesalius (1543). (Plate III. & IV.) It is hard to say whether the text or the illustrations exercised the stronger influence over the contemporaries and followers of Vesalius, but the very number of his imitators serves to prove the greatness of this influence. Apart from the internal evidence which Vesalius' magnificent work on anatomy provided there would be little else to show that his interest in anatomy extended to the æsthetic arts, were it not for one other fact, namely, he numbered among his friends Titian, who "for the pictorial representation of nature surpassed all other great painters of Italy."

One of Titian's pupils, Johan Stephan von Calcar, executed the plates which illustrate Vesalius' anatomy. Calcar painted the portrait of Vesalius which is one of the treasures of the Royal College of Physicians in London.[1]

Thus the fashion arose of employing admirable designers to illustrate works on anatomy. But a clear distinction must be made between the use of the æsthetic arts for purposes of illustration and record and the value of artistic powers of perception to the individual scientific observer. The application of art to illustrate medicine can only preserve and represent what is known, it does not discover the unknown. The art of medicine gained relatively little from the addition of artistic illustrations to the printed books. We see this exemplified in the numerous anatomical plates which came into vogue after Vesalius had shown what was possible. In his plates the illustration is secondary to the

[1] There is a folio volume entitled *Notomie di Titiano*, containing seventeen plates engraved by Bonavera. These plates are identical with Caspar's illustrations to Vesalius, except for being reversed. The title-page includes a head and neck of Titian copied from Caspar's portrait. I have failed to discover why Bonavera ascribed these seventeen plates to Titian. (Plate V.)

LAURENTIUS PHRIES, OR FRIES, *circa* 1500.

From the *Spiegel der Artzney*, Strasburg, 1518.

PLATE IV.

HVMANI CORPORIS OSSIVM CAETERIS QVAS SVSTINENT PARTIBVS LIBERORVM, SVAQVE SEDE POSITORVM EX latere delineatio.

VESALIUS,

1514–1564.

From the *De Corporis Humani Fabrica*, Basle, 1543.
Woodcuts by Caspar

[TITIAN ?]

1477–1576.

From the *Notomie di Titiano.*
17 Plates engraved by Domenicus
Bonavera. (No text, no publisher,
no place, no date.)

CHARLES ESTIENNE (CAROLUS STEPHANUS), 1503–1564.

From the *De Dissectione Partium Corporis*, Paris, 1545.

subject of the text, at the hands of his successors the anatomy becomes little more than the vehicle for the fanciful designs of the draughtsman. (Plate VI.) As artistic compositions the illustrations are striking, but the anatomy is grotesque. Even Bidloo's plates did not add largely to science ; he composed some bad text for the highly-praised plates of the artist Gérard de Lairesse. (Plate VII.)

As time went on the printing press and its ancillary process of mechanically reproduced illustrations made great strides towards perfection, so that good illustrations eventually became a matter of course. Yet the onward progress of medicine was fitful. It seems as though important discoveries were only possible when the exceptional man appeared in whom the artistic sense was united with the scientific spirit. All primary discoverers are artists in the sciences they work in.

Such was William Harvey, who in 1628 published his immortal treatise on the circulation of the blood. The printed book owed nothing to the indifferent copper plates that illustrated it. What was it that this round-faced, olivaster-complexioned man possessed which enabled his eye, "very black and full of spirit," to see far more clearly than his fellows ? Harvey's genius lay in his inimitable powers of artistic perception, not used for creative art or for mere illustration, but applied rather to accurate observation. To those powers he added imagination wherewith to frame hypotheses, and patient judgment to test his hypotheses by investigation and experiment, whilst we know from the records of his life that he was likewise a keen student and critic of the fine arts. When he went on his diplomatic journey with the Earl of Arundel through Europe a member of the Embassy wrote a letter speaking of "honest little Harvey whom the Earl is sending to Italy about some pictures for his Majesty." I wonder what those pictures were? "His Majesty" was King Charles I., who brought the Leonardo MS. on anatomy to the Royal Library now in Windsor. Nothing more is known of how it came into the King's possession. The present librarian has courteously given me this information. Can Harvey have secured it, with the "pictures for His Majesty"?

More than once in his writings the artist's mind shows itself in the man of science. In his *De Generatione* Harvey compares the artist and the scientist :—

"The things that have formerly been noted, and that by use or wont have become firmly fixed in the mind of the artist, do, in fact, constitute art and the artistic faculty: art indeed is the reason of the work in the mind of the artist.

"On the same terms, therefore, as art is attained to is all knowledge and science acquired; for as art is a habit with reference to things to be done, so is science a habit in respect of things to be known. Each has its origin in sense and experience, and it is impossible that there can rightly be either art or science without visible instance or example."

Harvey lived to a good old age, and died in 1657 in his 80th year. Thomas Willis was then 35 years old. He was one of the little group of savants to whom we owe the foundation of the Royal Society. Willis followed the example of Vesalius, and sought the help of one of the greatest draughtsmen of his time, Christopher Wren, to illustrate his anatomy of the brain. (Plate VIII.)

Wren, however, was no mere illustrator of anatomical treatises. During his residence at Oxford he pursued his scientific studies, and in anatomical science he stood among the first professors of the day. He acted as anatomical demonstrator at the lectures and dissections of Scarborough and Willis.

Moreover, Wren was the author or discoverer of the

Godofridus Bidloo,

1649-1713.

From the *Anatomia Humani Corporis*, Amsterdam, 1685.
Plates by Gérard de Lairesse.

THOMAS WILLIS,

1622–1675.

From the *Cerebri Anatome*.

Plates by Sir Christopher Wren.

anatomical experiment of intravenous injection. He injected wine and ale into dogs' veins and made them drunk; he also experimented with opium, scammony and other drugs, showing that they exerted their proper effects when thus injected. These experiments he hoped might bring great light to the theory and practice of physic. Among his anatomical works are to be found descriptions of the bones of the arms, dissection of the eel and experimental splenectomy in the dog. If the rebuilding of London after the Fire of 1666 had not brought him greater fame, we might still have held him in high honour for his anatomical and physiological researches, nor have forgotten that when Glisson, Wharton and Hodges stayed out the Plague in London Wren faced it with them. In February, 1923, we shall celebrate the 200th anniversary of Wren's death, and I trust that the medical profession will pay fitting tribute to a worthy exemplar.

Medicine in the eighteenth century was remarkable chiefly for the rise of innumerable systems and sects. Philosophy and metaphysics were the vogue; scientific observation such as Harvey knew was less fashionable.

In the midst of a welter of Eclecticism, Animism, Vitalism, Mechanico-dynamism, Hoffmanism, Magnetism, Realism, and Brunonianism there came a modest Viennese physician, "a great friend of music and the arts in general."

Auenbrugger was born on Nov. 19th, 1722, just 200 years ago. He made the simple observation "that the chest of a healthy man resounds when struck." "I lay before you, benevolent reader," he says, "a new sign for the elucidation of diseases of the chest. . . . This consists in the percussion of the human thorax, by the varying resonance of whose tones a judgment may be formed as to the internal condition of this part."

Recollect that prior to this announcement only one physical sign of any thoracic disease had been described, nothing had been added to the "succussion splash" of Hippocrates. Auenbrugger's brilliant suggestion laid the foundation for our whole method of physical examination of the chest.

It is singular to note how little medicine owes to the "systems" which men have invented for its study and practice, how much it owes to apparently disconnected observations. Philosophy and metaphysics have added nothing to our knowledge and understanding of medicine.

Auenbrugger in his spare moments dabbled in music, he even composed an opera (*The Chimney-sweeps*), which was performed, without marked success. I am not sure that it has survived to the present day. But his love and understanding of music enabled him to discover the art of percussion, and have given to posterity more lasting benefits than all the operas of past and present times.

In England the eighteenth century produced men endowed with similar faculties, notably the two brothers William and John Hunter, both of whom displayed the keenest appreciation of the arts. William was the first professor of anatomy to the Royal Academy, and published a treatise on the "Gravid Uterus," upon the plates of which the foremost engravers of the day were employed. John Hunter, whom as an anatomist men have put equal with Vesalius, had a large share of his love of art. In his collection were to be found pictures by Teniers, Ostade and Vandevelde. His friendship with Sir Joshua Reynolds is well known, and gave us the magnificent portrait of Hunter that Sir Joshua painted.

In the series of letters that John Hunter wrote to Jenner there are many references to the purchase of pictures, for these two friends were not only united by their common interest in natural history, their love of artistic things was

PLATE IX.

SIR CHARLES BELL,

1774–1842.

A soldier wounded at Waterloo. (Pencil drawing in possession of the Bristol Medico-Chirurgical Society.)

a further bond. In 1776 or 1777 Hunter writes to Jenner: "I must pick you up a picture this winter." And again: "Pictures have been very cheap, but the season is now over. There will be but one sale, viz., Fordyce's; but I believe all his pictures are exquisite and will go beyond you or me."

Jenner himself, the incomparable country doctor, is the aptest figure of all to illustrate my theme. Was it not his artist-eye that, falling upon the unscarred faces of the Gloucestershire dairy-maids, discovered that they owed the preservation of their good looks to the cow pox? Baron's life describes how "he had the keenest relish for picturesque beauty, and in his excursions alike gratified his taste in this respect and increased his knowledge by pursuing the details of natural history"; and elsewhere says, "Those who have accompanied him for 20 or 30 miles in a morning listened while with a vivid and imaginative fervour he shadowed forth his own feelings or with a painter's eye and poet's tongue delineated the beauties around him."

Such a man, too, was Sir Charles Bell (born 1774), who took charge of a hospital in Brussels after the Battle of Waterloo, and made the pencil sketch of a wounded soldier which our Society so jealously prizes. (Plate IX.)

In 1816 he published his momentous discovery that the posterior roots of the spinal nerves carry the sensory fibres and the anterior roots the motor, a discovery as important to the knowledge of the nervous system as Harvey's to the circulatory. Bell wrote in 1821 of his discovery, "I know this will put me beside Harvey." Even without this epoch-making observation his studies on the anatomy of expression would have entitled him to a high place among the great scientists who were artists.

This year marks the centenary of the birth of another master-artist in science. Pasteur was born in 1822. At school he was a merely average pupil. He showed a rather marked taste for drawing. A few successful efforts in chalks and in charcoal seemed to foretell for him an artistic career.

Fortunately for science, and medicine in particular, Pasteur found in the study of nature more complete play for his æsthetic and artistic sensibilities than in continuing along the paths of creative art. It was the study of crystals and the science of crystallography that first attracted him into natural science. To him perhaps Paget's remarks in his Hunterian address apply, " I cannot doubt that in the contemplation of the order and mutual fitness in the great field of scientific truth there may be, to some high intellects, a source of pure delight such as are the sensuous beauties of nature to the cultivated artist-mind."

Although Sir James Paget was speaking of John Hunter, there can be little doubt that he was expressing sentiments which he had experienced to the full. Paget, like Pasteur, had shown considerable talent for drawing at an early age, throughout life he was no mean judge of music and painting, like John Hunter he made some of his closest friends amongst artists. I have found in the *Memoirs and Letters* of Sir James Paget his own estimate of the value of the fine arts:—

" In close connection with the study of natural history was that of some small measure of the fine arts. My father was a friend and patron of Old Crome. . . . Pictures, engravings, drawings were everywhere in the house: and art and artists were talked of, and Young Crome succeeded Old Crome in his weekly visits at the house, and nearly all of us had lessons from him." He goes on to say that the immediate utility of this meagre education in painting and drawing was little, but " its indirect utility was too great to be told. It helped to enable me to look and see more in

PLATE X.

SIR JAMES PAGET,

1814–1899.

Woolsey's Mill, near Yarmouth.
(From a pencil sketch by Paget during Apprenticeship.)
*Block lent by Messrs Longmans, Green & Co.*

J. GREIG SMITH,

1854—1897.

Medallion of Dr. E. Long Fox.
(In the possession of Mrs. P. Watson-Williams.)

things than some could see: it strengthened the power of remembering things seen." (Plate X.)

Innumerable additions might be made to the instances I have quoted. There is Graves, whom Trousseau described as "a perfect clinical teacher, observer, philosopher, ingenious artist"; and Addison, who superintended the making of the wax models in the museum of Guy's Hospital. In our own city Greig Smith, one of the founders of abdominal surgery, was no mean modeller in clay, as his medallion portrait of Dr. Long Fox testifies. (Plate XI.)

But amongst all the moderns Charcot must surely stand first. The unrivalled method of studying nervous diseases which he instituted at the Salpêtrière was the outcome of an artistic genius closely akin to that which inspired the Greeks. Henri Meige, writing of him in 1898, speaks of "the artist who, in Charcot, went hand in hand with the physician, so that under his influence an artistic spirit came into being at the Salpêtrière which reflected an unwonted lustre on medical science."

To conclude, what is the upshot of these reflections on the importance of the artistic sense to scientific progress as a whole and to the science of medicine in particular?

Galton in his enquiry into the power of mental imagery came to the conclusion that scientific men as a whole did not see with the mind's eye. He found scattered instances amongst men of science, though by no means in the same abundance as amongst the generality of people untrained to science. In youth the proportion who exhibit this faculty is high, but the study of languages and book-learning tends to dull it. The delight of recalling beautiful scenery and great works of art is one of the highest pleasures. Those who possess the power carry whole picture galleries with them. Moreover, this ability to recall by mental visualisation transcends far the possession of carefully registered notes on the shelves of a well-stocked library.

Unfortunately, a habit of suppressing mental imagery seems to characterise men who deal with abstract ideas, and men of abstract ideas are often looked upon as likely pioneers in science. So that this faculty, which is so important in all technical and artistic occupations, that gives accuracy to our perceptions and justness to our generalisations, runs a risk of being suppressed by our bookish and wordy education, and of being deliberately starved by the lazy disuse of philosophers.

Children, as Galton discovered, have far stronger powers of visual representation than scientific men as a class. The basis of accurate mental imagery lies in keen and rapid perception by the senses. Memorising by rote and from the printed page, or worse still by some jingle of words, is a poor substitute for recollection by means of artistic receptivity, which seems to be nature's endowment of nearly every child. The student of medicine cannot afford to allow this gift to be deliberately starved by philosophical neglect or belittled by the contempt of pedants, for it seems to me that one of the chief factors which has transformed the healing art from mere magic into scientific medicine has been the happy conjunction of the artist and the scientist in the same individual.

That one man should combine these two faculties developed to a high level is uncommon. When such a singular genius arises he seems to move in an orbit of his own, he seems not to belong to the period of the world's history in which he lives.

His discoveries are scarcely influenced by the age in which they are made. Lapse of years cannot impair their freshness. What historian of philosophical method could assign to their proper date on internal evidence Harvey's discovery of the circulation of the blood, or Bell's discovery of the motor and sensory nerve roots ?

A certain inevitable progress in knowledge can be made by those drudges who can dig with labour and patience even though they have not wings to fly. They can follow the road and reach new milestones, but they are always tied to the road. "Art," as John Brown phrased it, "can go across country."

It is, I believe, the artistic sense that raises the scientist above the drudge and furnishes him with wings to fly.

## Figures and Tables

**Figure f1:**
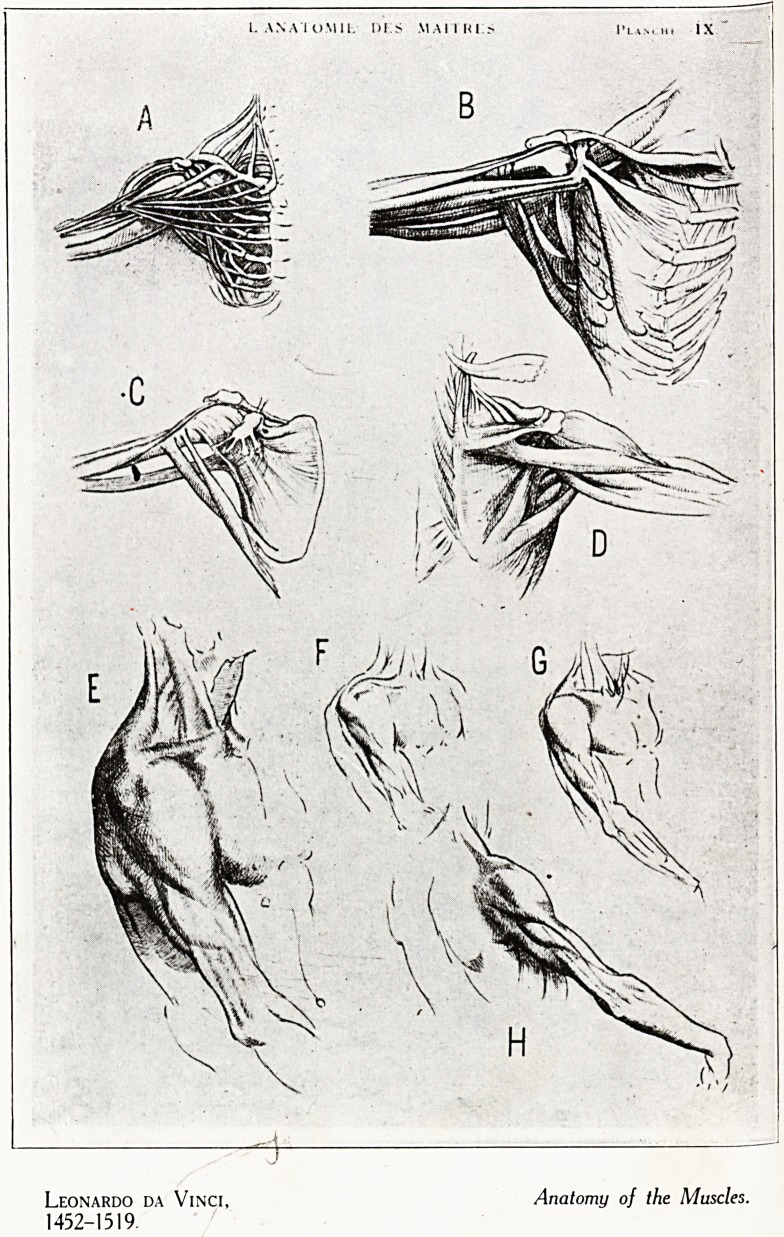


**Figure f2:**
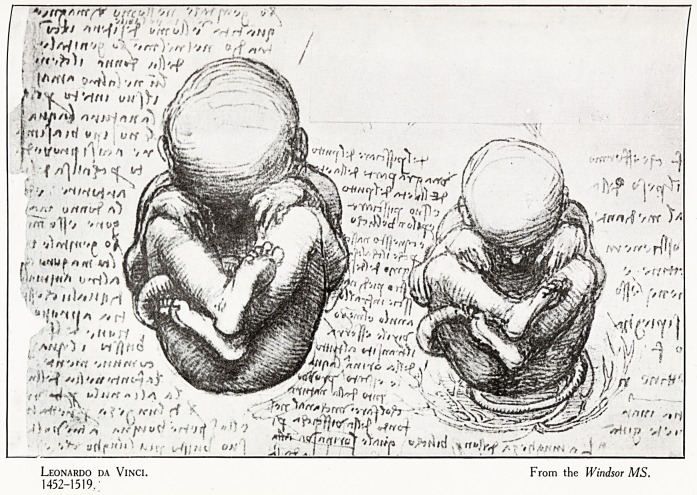


**Figure f3:**
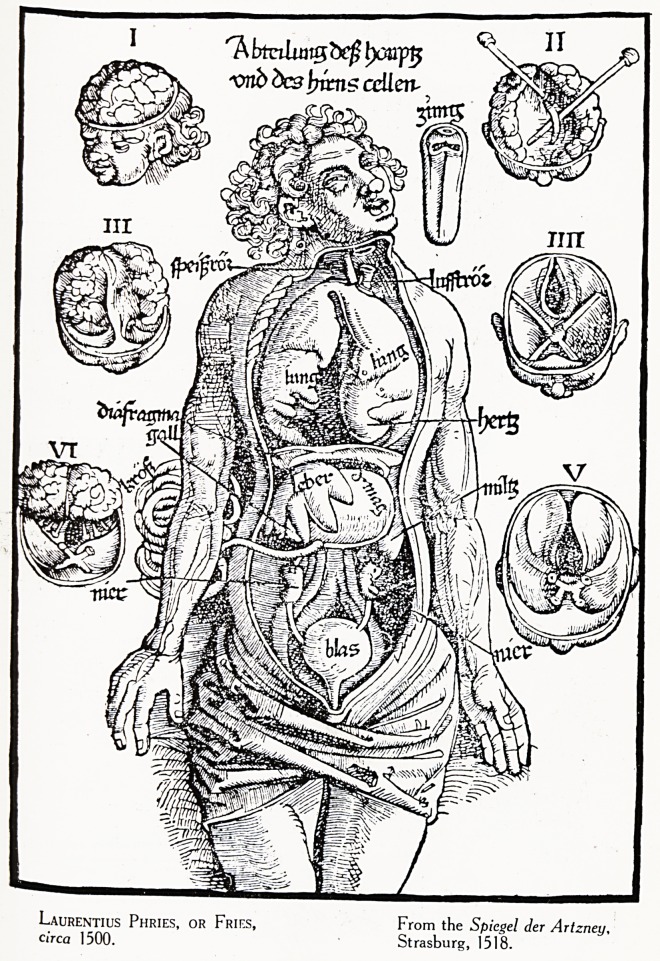


**Figure f4:**
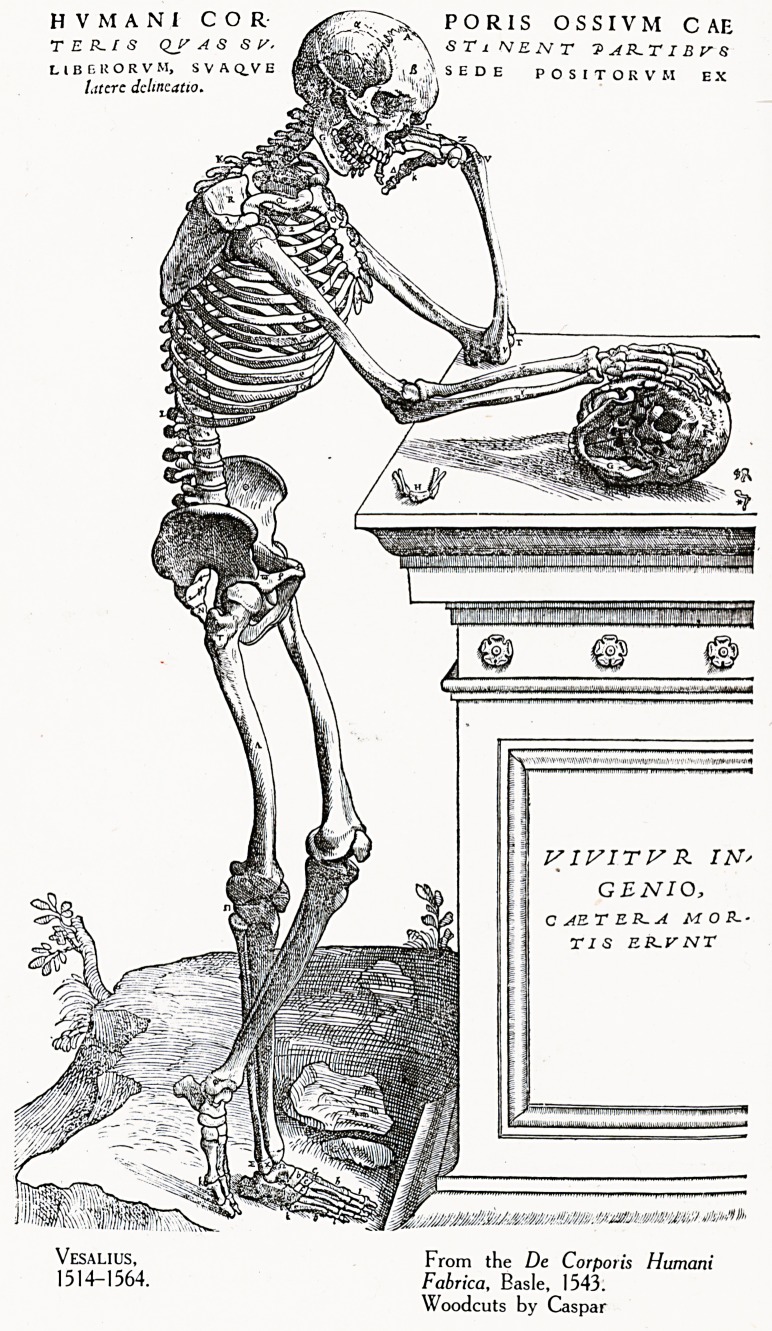


**Figure f5:**
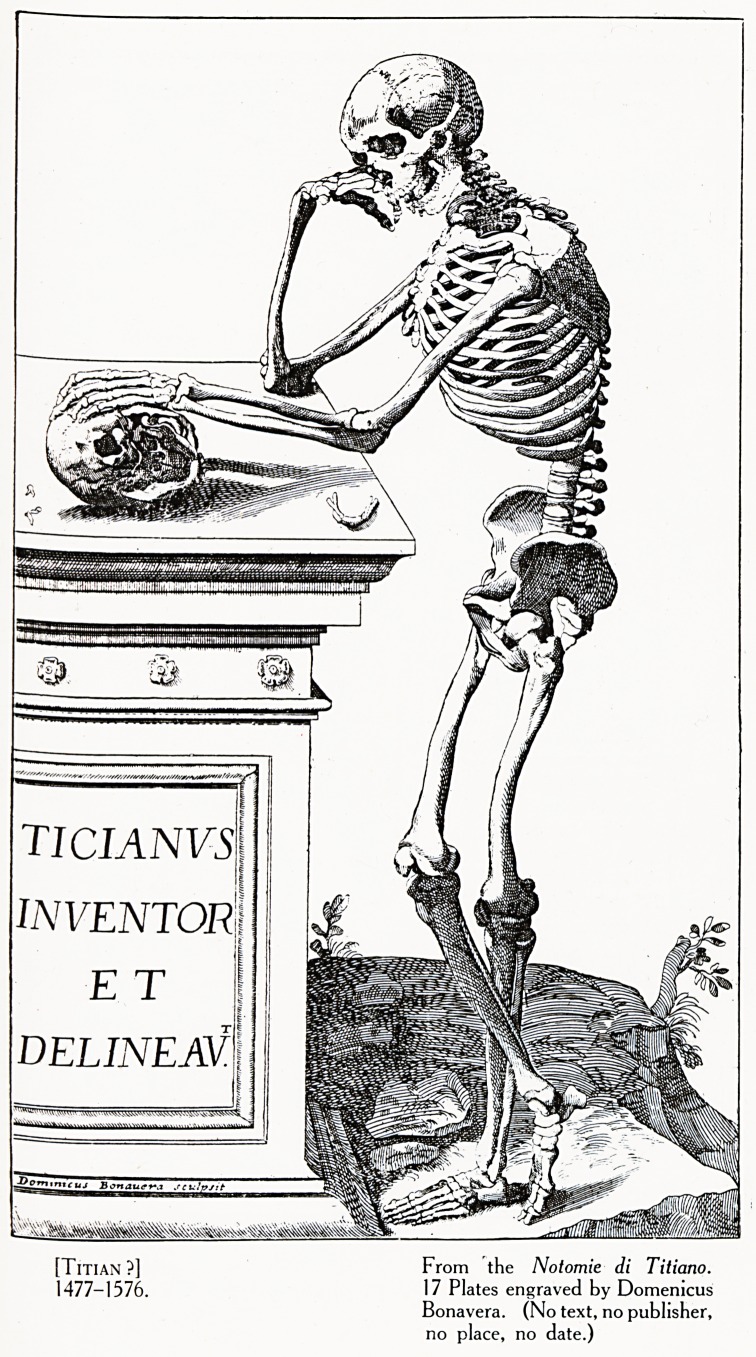


**Figure f6:**
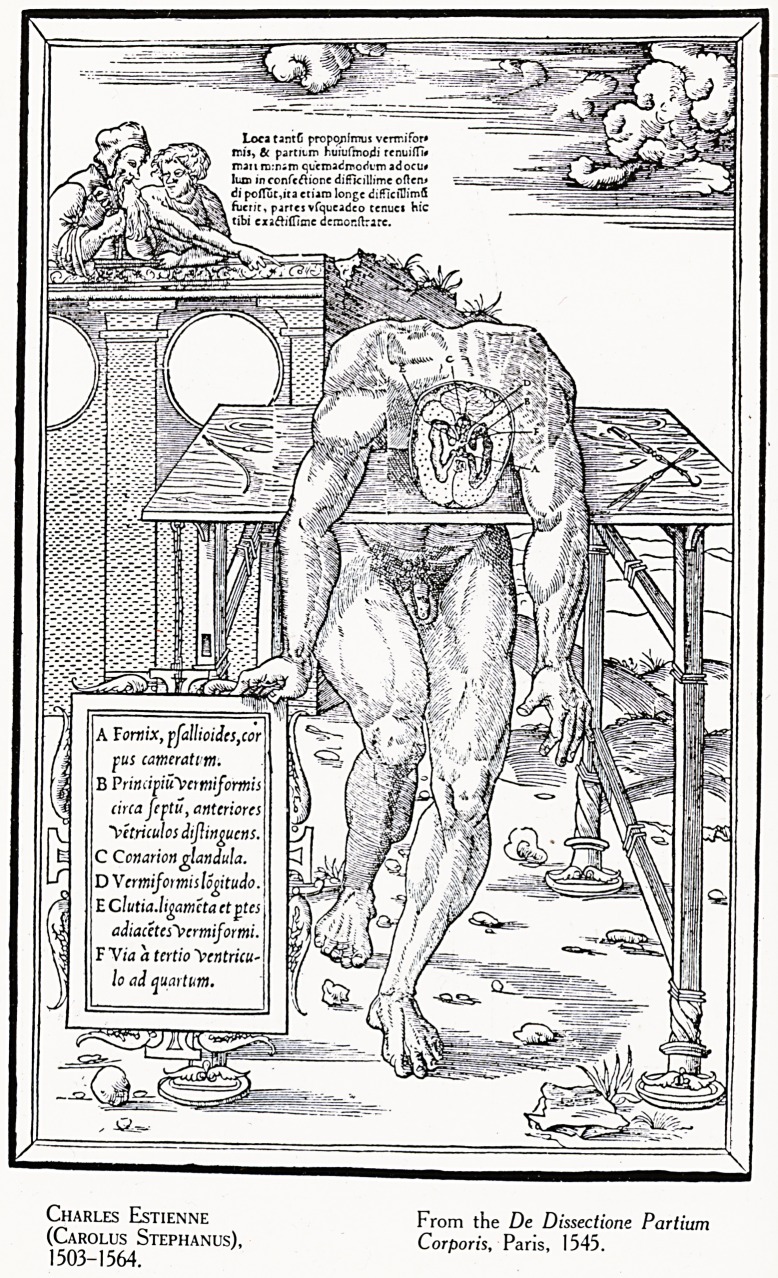


**Figure f7:**
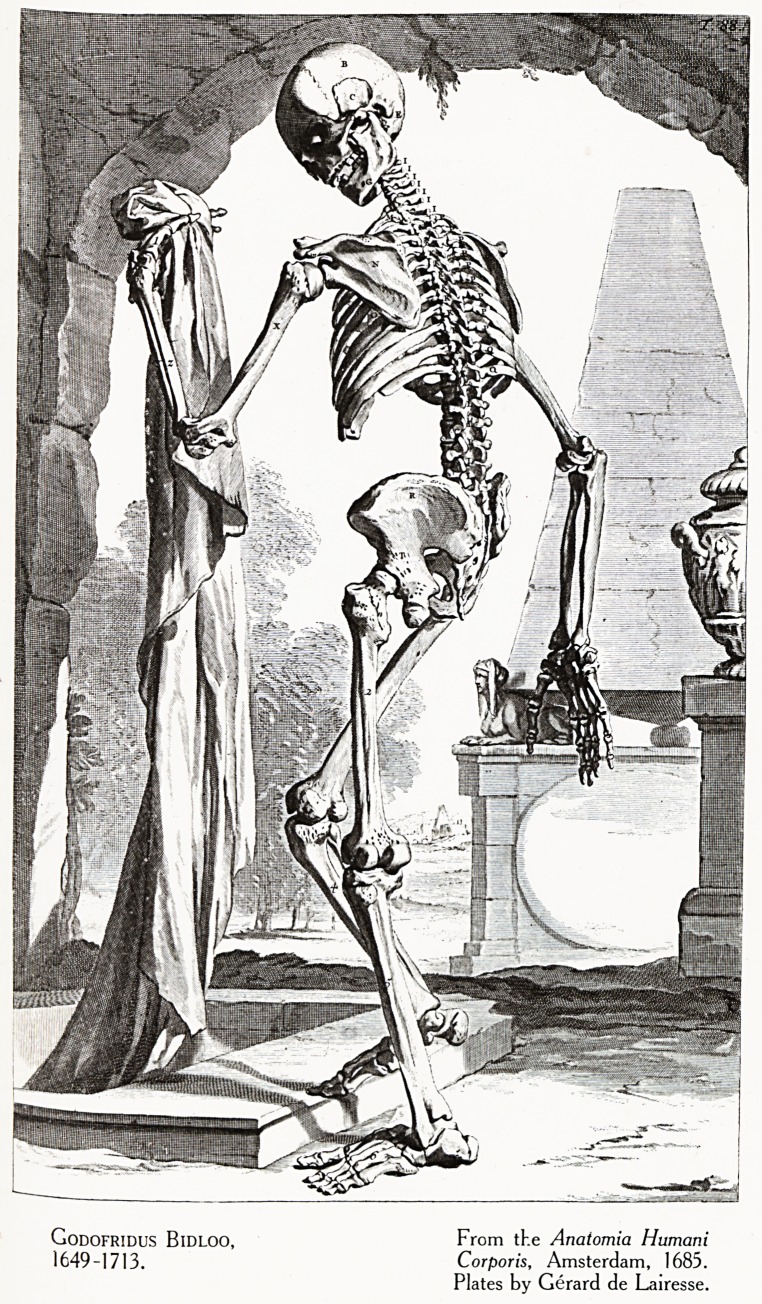


**Figure f8:**
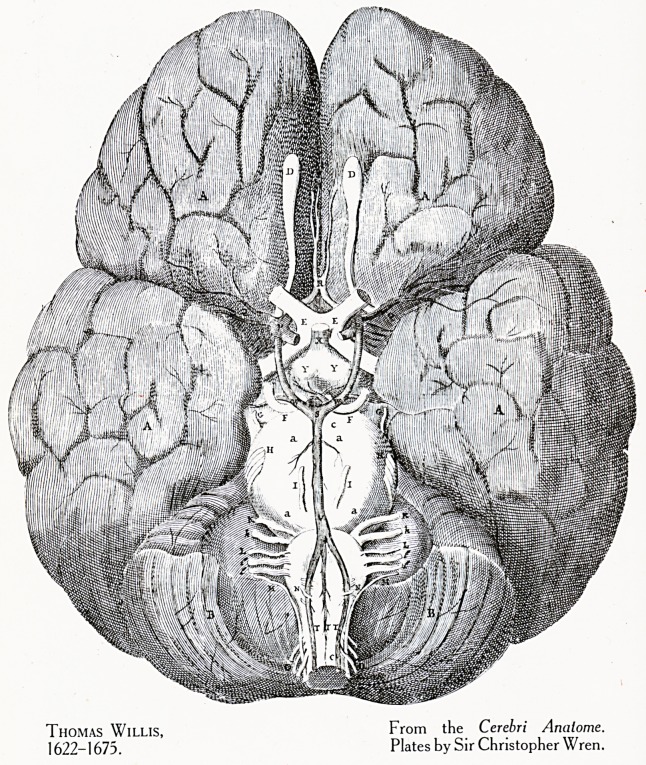


**Figure f9:**
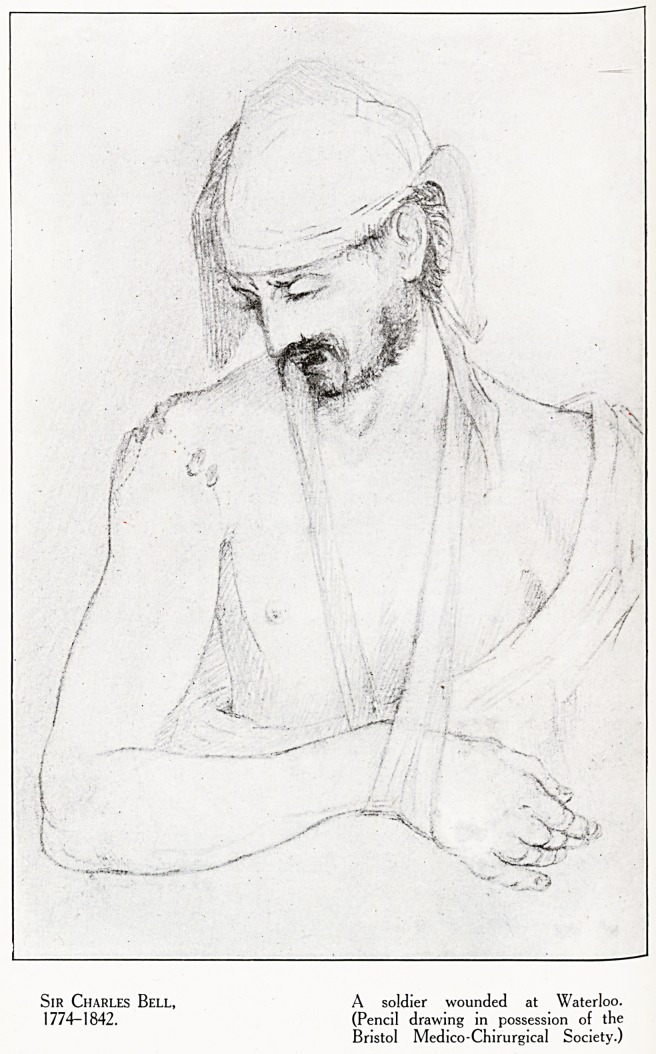


**Figure f10:**
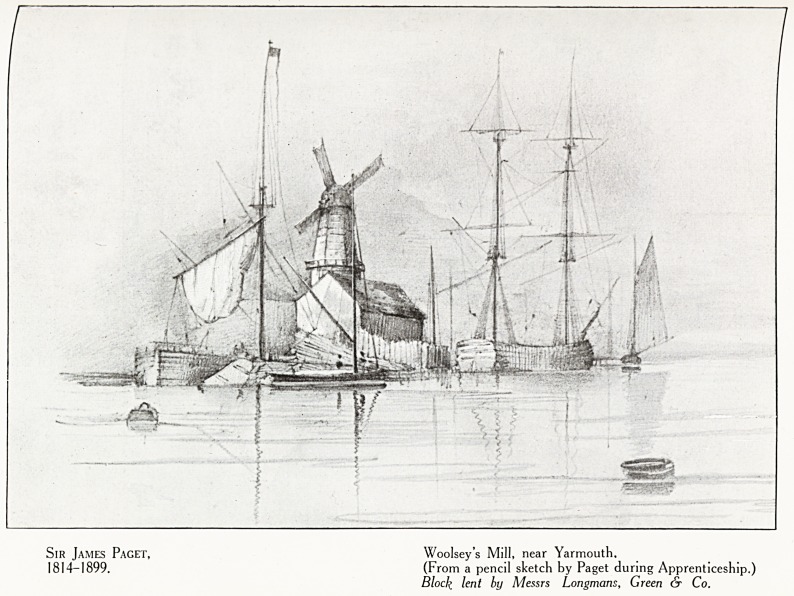


**Figure f11:**